# Recent Advancements of Transcranial Direct Current Stimulation and Machine Learning: Methods, Challenges, and Opportunities

**DOI:** 10.53941/tai.2026.100005

**Published:** 2026-03-05

**Authors:** Junfu Cheng, Tara Sahni, Zeyun Zhao, Skylar E. Stolte, Chenyu You, Adam J. Woods, Aprinda Indahlastari, Ruogu Fang

**Affiliations:** 1. Department of Electrical and Computer Engineering, Herbert Wertheim College of Engineering, University of Florida, Gainesville, FL 32611, USA; 2. Rutgers Center for Cognitive Science, School of Arts and Sciences, Rutgers University-New Brunswick, New Brunswick, NJ 08854-8020, USA; 3. J. Crayton Pruitt Family Department of Biomedical Engineering, Herbert Wertheim College of Engineering, University of Florida, Gainesville, FL 32611, USA; 4. Center for Cognitive Aging and Memory, McKnight Brain Institute, University of Florida, Gainesville, FL 32611, USA; 5. Department of Clinical and Health Psychology, College of Public Health and Health Professions, University of Florida, Gainesville, FL 32611, USA; 6. Department of Applied Mathematics & Statistics, College of Engineering and Applied Sciences, Stony Brook University, Stony Brook, NY 11794, USA; 7. Department of Computer Science, College of Engineering and Applied Sciences, Stony Brook University, Stony Brook, NY 11794, USA; 8. School of Behavioral and Brain Sciences, The University of Texas at Dallas, Richardson, TX 75080, USA

**Keywords:** transcranial direct current stimulation (tDCS), machine learning, precision neuromodulation

## Abstract

Transcranial direct current stimulation (tDCS) has emerged as a versatile non-invasive neuromodulation approach that can alter cortical excitability and affect network plasticity. Recent advances in machine learning (ML) offer an opportunity to transform tDCS from largely heuristic practice into a quantitatively informed, adaptive intervention paradigm. Here, we synthesize developments from 2020 to 2025 at the intersection of tDCS and ML. Search results from structured PubMed and Google Scholar queries were screened for eligibility based on predefined inclusion criteria, retaining peer-reviewed studies that applied ML techniques to tDCS related studies. Eligible studies were evaluated for data integrity, and ML model validation methodology. Sixteen studies met inclusion criteria. Across these studies, ML was applied to heterogeneous datasets, including electroencephalography, neuroimaging, and clinico–demographic features, to predict stimulation outcomes, characterize neural responses, and identify biomarkers of tDCS sensitivity. Support vector machines and random forests remain prevalent, reflecting the modest scale and exploratory nature of current datasets; most studies rely on early-stage clinical or preclinical cohorts, resulting in promising yet fragmented evidence. Nevertheless, emerging results illustrate how ML can reveal latent physiological structure, guide dose–response optimization, and support the translation of tDCS toward precision neuromodulation. Drawing on this integrated analysis, we highlight key directions for the field: multimodal integration that unifies electrophysiological, structural, and behavioral signatures; incorporation of biophysically grounded forward models and pretrained deep-learning architectures; and development of adaptive, closed-loop control strategies capable of personalizing stimulation in real time. Together, these advances chart a pathway toward ML-guided tDCS systems that are mechanistically informed, clinically actionable, and scalable for widespread application.

## 1. Introduction

Transcranial Direct Current Stimulation (tDCS) is a non-invasive neuromodulation technique. It is one of three main transcranial electrical stimulation (tES) methods, alongside transcranial alternating current stimulation (tACS) and transcranial random noise stimulation (tRNS). Guleyupoglu et al. [1] provided a full classification of tES techniques.

Clinical trial studies have shown that tDCS is a promising technique for influencing neural activity, cognitive processes, and behavioral and psychiatric outcomes, making it an increasingly valuable tool in neuroscience research, as indicated by Chase et al.’s review [2–6]. Over the past decade, tDCS has been investigated for its potential therapeutic applications in various neurological and psychiatric conditions, such as cognitive enhancement, stroke, and depression. The development history of tDCS clinical trial studies can be explored through databases such as the one available at tdcsdatabase.com. Recent meta-analytic evidence from Begemann et al. demonstrates that tDCS produces small but significant improvements in cognitive functions such as working memory and attention across neuropsychiatric disorders [7]. Sun et al.’s survey further shows that tDCS significantly enhances motor strength recovery in stroke patients, particularly during the acute and subacute phases [8]. Complementing these findings, Heiland et al. and Palm et al. report that tDCS reduces tinnitus severity and comorbid depressive symptoms, with Palm et al. noting the greatest antidepressant effects in non–treatment-resistant populations [9,10]. Together, these findings highlight the multifaceted therapeutic potential of tDCS across cognitive, motor, and affective domains.

Human and animal studies have shown preliminary evidence that tDCS affects brain function by subtly changing neuron activity and enhancing plasticity in brain networks. Clark et al.’s study found the enhancement of glutamatergic activity and neuronal metabolism in brain regions using ^1^H magnetic resonance spectroscopy in humans [11]. Fritsch et al.’s experiment on mice showed that anodal tDCS promotes Brain-Derived Neurotrophic Factor-dependent synaptic plasticity [12]. Fritsch et al. measured these changes using extracellular recording electrodes to detect field excitatory postsynaptic potentials (fEPSPs) in acute mouse motor cortex slices [12].

Giordano et al. summarized that, despite various theories having been advanced to explain tDCS mechanisms, no widely accepted framework explains how tDCS works [13]. Giordano et al. found that many of these tDCS clinical trial studies rely on a simplistic assumption, placing electrodes over target brain areas and expecting polarity-based effects (where anodal electrodes are assumed to increase cortical excitability at the targeted brain regions, whereas cathodal electrodes are assumed to decrease it) [13]. However, Giordano et al. criticized that this simplistic assumption overlooks key complexities such as dose effects, brain current flow physics, the nuanced link between brain activity and behavior, and the oversimplified view that cognitive function and disorders are tied to single brain regions [13]. Characteristics of the electrodes, such as electrode size, shape, material composition, and placement, directly influence current density, stimulation focality, and user comfort based on Solomons et al.’s review [14].

According to the summary by Li et al., effects of tDCS vary with individual traits (e.g., age, genetics, neuroanatomy, baseline cognitive/motor ability, psychological state, neurochemical profile), brain states (e.g., task-related activity, circadian rhythms, cortical excitability, neurotransmitter levels), and stimulation parameters (e.g., current intensity, duration, electrode size, montage, and timing relative to tasks) [15].

Research in tDCS still faces important gaps, highlighting the need for studies to (1) determine the optimal dose for each individual, (2) identify the most effective brain regions and networks to target, and (3) establish reliable biomarkers for monitoring treatment outcomes. One critical component in addressing these challenges is understanding why individuals respond differently to stimulation, which may help explain inconsistencies across study results. This is particularly important since most traditional tDCS clinical trials still rely on a one-size-fits-all intervention protocol that may not account for individual variability. To bridge the divide between basic science and clinical application, theoretical modeling and animal studies play a vital role in informing and guiding human research.

To address these key gaps in tDCS research, comprehensive measurement and theoretical modeling are essential. Neuroimaging, electroencephalography (EEG), and clinico-demographic data help uncover biomarkers, monitor treatment effects, and explain why responses differ across individuals. These tools are also crucial for translating basic science and theoretical models into effective clinical applications.

Neuroimaging is crucial in tDCS studies by examining brain structures like cortical thickness and surface area. These neuroanatomical features can serve as biomarkers to predict who is likely to benefit from tDCS, supporting a shift toward personalized interventions. By linking brain anatomy to behavioral outcomes, neuroimaging enables more accurate case-level predictions and helps bridge the gap between brain structure and cognitive function [16].

EEG offers opportunities to monitor and understand brain activity changes in tDCS studies for psychiatry and cognitive ability. Moraes et al. assessed rapid eye movement (REM) sleep brain activity changes induced by donepezil, and found that donepezil treatment in patients with Alzheimer’s disease (AD) increased REM sleep and reduced slow-frequency activity during REM sleep, and that higher REM sleep alpha power was strongly associated with greater cognitive improvement [17]. Babiloni et al. found that in mild cognitive impairment and AD, lower gray matter volume was linked to higher delta activity and lower alpha activity in resting-state EEG [18]. By analyzing these patterns, researchers can better understand the underlying neurophysiology of AD and evaluate how interventions like tDCS may alter brain function. For example, in a pioneering tDCS study, Andrade et al. used EEG to (1) measure brain activity changes induced by multisite anodal tDCS + cognitive stimulation in AD, and (2) link these neural changes to improvements in cognitive performance [19]. In addition to neuroimaging and EEG, clinico-demographic data records are also valuable features to explore, as they can provide important context and improve the prediction of individual treatment responses.

The development of Machine learning (ML) or deep learning (DL) provides clear advantages for tDCS research. These ML/DL techniques have spanned disease diagnosis, brain imaging analysis, and brain-computer interfaces [20–25]. As highlighted by Pouyanfar et al., ML is particularly effective in handling high-dimensional, complex, and noisy data, which are common in neuroscience [26]. When inferential statistics are essential in clinical trial analysis to draw conclusions about treatment effects and determine their significance, generalization is a key advantage of ML over inferential statistics. ML focuses on building models that generalize well to new, unseen data using training data as a guide to manage nonlinear relationships and extract meaningful patterns from large, heterogeneous datasets. Applying ML to tDCS enables researchers to identify patterns in neural responses, optimize stimulation protocols, and develop predictive models for individual outcomes.

ML models can analyze multimodal data such as neuroimaging, electrophysiology, and behavioral measures, to (1) improve the personalization of stimulation, (2) enhance our understanding of the neural mechanisms underlying tDCS effects, and (3) support real-time analysis and adaptive protocols, which are critical for advancing tDCS from experimental settings to practical clinical applications.

Although research on tDCS and ML is expanding, there are few comprehensive reviews. The goal of this review is to comprehensively examine and synthesize recent efforts to integrate tDCS studies with ML, with the aim of enhancing personalization, optimizing stimulation protocols, and enabling predictive modeling for improved tDCS intervention outcomes across diverse therapeutic indications. This includes systematically identifying the main application domains, commonly used data modalities, and ML techniques, and proposing future research directions to advance precision neuromodulation.

Taken together, our survey makes three primary contributions. First, we map the integration of tDCS and ML across clinical, cognitive, and computational domains. Second, we distill the methodological strengths and weaknesses of current approaches, covering data types, modeling choices, and evaluation practices. Third, we propose a forward-looking agenda for developing more precise, multimodal, and adaptive tDCS systems driven by ML. Together, these contributions aim to advance precision neuromodulation toward more effective and individualized stimulation strategies.

## 2. Method

This section describes the methodology used to conduct a review of studies integrating tDCS and ML. It outlines the search strategy, study selection criteria, and procedures for data extraction and evaluation.

### 2.1. Search Strategy

**Figure 1.**
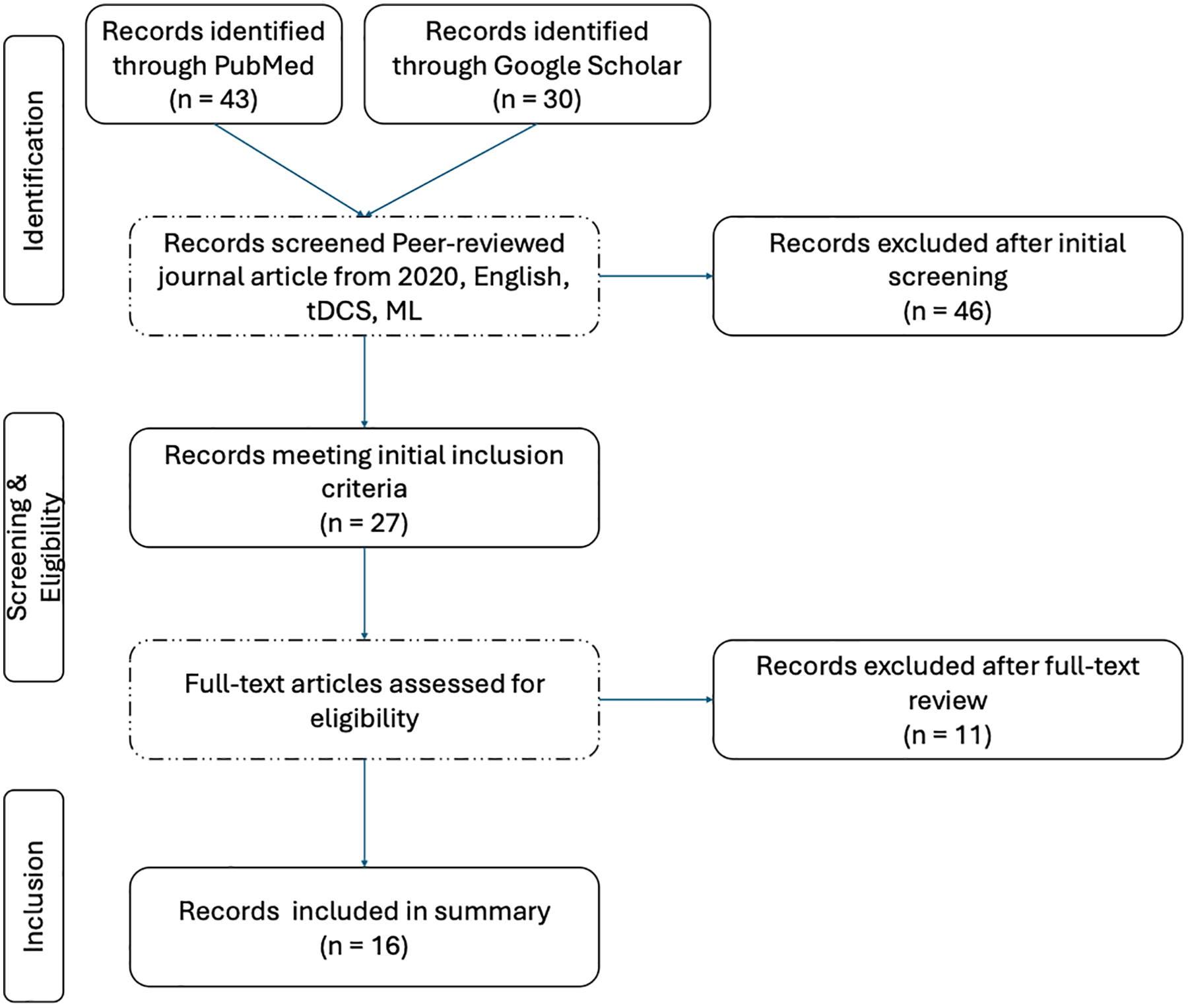
Flowchart illustrates the systematic search and screening process for selecting relevant tDCS and machine learning studies from PubMed and Google Scholar. After removing duplicates and excluding articles based on type and language, 27 papers remained from both databases. After additional eligibility screening that excluded surveys and unrelated studies, 16 papers remained and were comprehensively summarized.

The primary objective was to review studies integrating tDCS and ML and to identify and characterize their major application areas. The lead author independently assessed all studies to determine whether they met the predefined inclusion criteria. The study selection process is summarized in the flowchart shown in Figure 1. A systematic search was conducted in the PubMed database first. The following criteria were applied. Inclusion criteria: peer-reviewed journal articles published from January 2020 to May 2025, written in English, human studies, and focused on applications involving both tDCS and ML. Exclusion criteria: non-peer-reviewed articles, conference abstracts without full texts, studies lacking either tDCS or ML components, and review articles. The following keywords and Boolean search terms were used:

(“Transcranial Direct Current Stimulation” [Title/Abstract] OR “tDCS” [Title/Abstract]) AND (“Machine Learning” [Title/Abstract] OR “Deep Learning” [Title/Abstract] OR “Artificial Intelligence” [Title/Abstract]) AND (Optimization OR Personalization OR Brain Stimulation) AND (“2020/01/01” [Date-Publication]:”3000” [Date-Publication])

We also conducted a complementary search in Google Scholar using a year range of 2020–2025 to broaden the scope of the review. The following keywords were used in various combinations:

“Transcranial Direct Current Stimulation” OR “tDCS” AND “Machine Learning” OR “Deep Learning” OR “AI” AND “Brain Stimulation” OR “Optimization”

Given the large number of records retrieved, Google Scholar results were screened sequentially by relevance, and the process was stopped when two consecutive pages yielded no newly retained studies after title and abstract screening.

### 2.2. Data Extraction

Extracted variables included the study objectives, tDCS stimulation protocols, dataset characteristics (participants’ demographics, clinical, EEG, and neuroimaging data), software tools used, ML methods, therapeutic indications, and primary tDCS findings.

We identified and evaluated studies that used ML for tDCS outcome prediction or for EEG or functional magnetic resonance imaging (fMRI) analysis related to tDCS. To assess the clinical translational potential of these interventions, we evaluated each study’s developmental maturity using the Food and Drug Administration (FDA)’ s medical device regulatory framework. Accordingly, each study was assigned to a corresponding device development phase. For each study, we also extracted key trial characteristics, including sample size and blinding protocols.

Finally, we explored the wider scope of tDCS predictive modeling and EEG/fMRI analysis by categorizing studies based on input modality and clinical condition. We prioritized extracting clinically meaningful insights derived from ML across these domains. By examining how ML models were interpreted in ML-based tDCS studies, we summarized interpretability findings and identified features and patterns that provide insight into tDCS-related neurophysiological mechanisms.

## 3. Results

Although the initial searches yielded 73 records, only 16 studies met full-text eligibility criteria after screening, reflecting the limited number of peer-reviewed human studies that explicitly integrate both tDCS intervention and ML methods within the defined scope. This section summarizes the key findings from the reviewed studies, outlining the distribution of research focuses, data sources, data modality, ML approaches, and participant demographics in the integration of tDCS and ML. It further examines the evaluation metrics, data quality, and software tools employed across studies to identify prevailing trends and methodological practices in the field. In addition, the methodologies of the reviewed works are synthesized, with particular attention to neuroimaging-based modeling and tissue segmentation. The section also analyzes feature extraction strategies applied in ML models and discusses the clinical findings among tDCS studies.

### 3.1. Study Characteristics, Devices, and Data Modalities

#### 3.1.1. Trends and Variability in tDCS Device Usage

**Figure 2.**
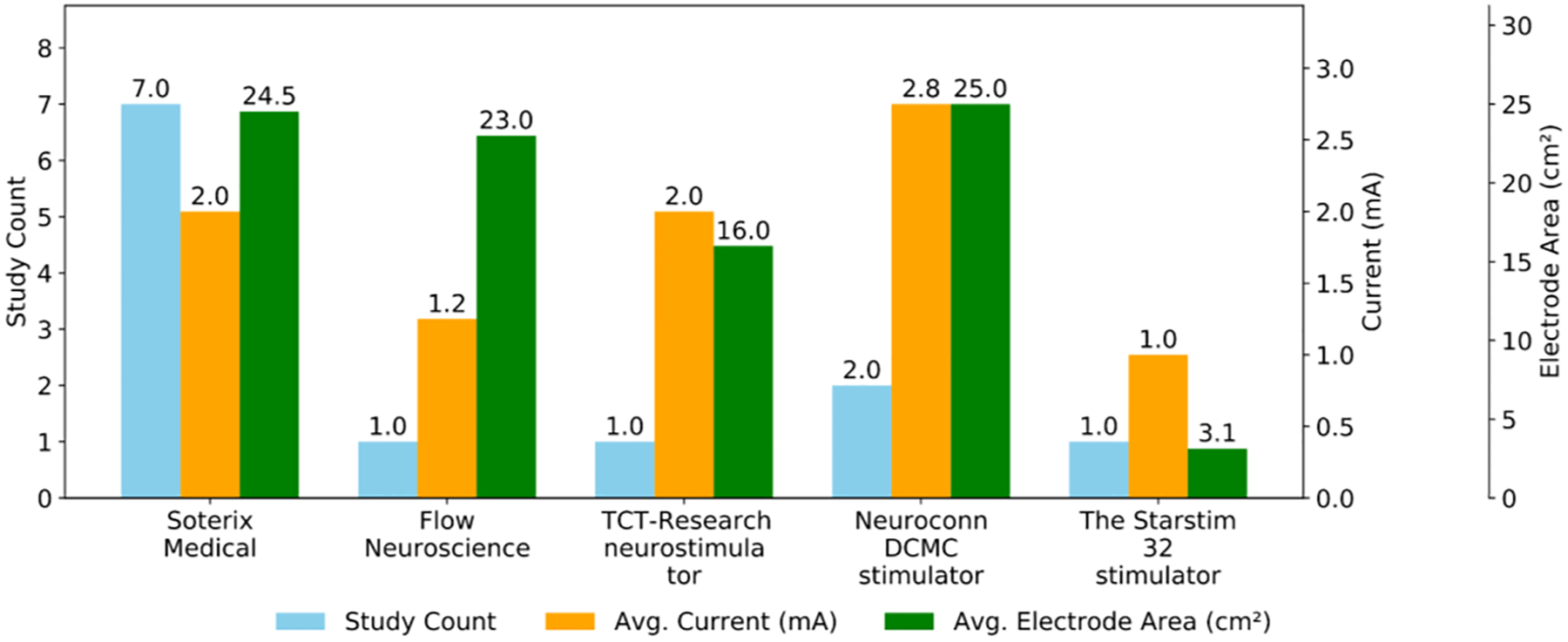
Comparison of tDCS devices by number of studies, average current intensity (mA), and average electrode area (cm^2^). Soterix Medical devices are the most frequently used, while current and electrode area vary across manufacturers, reflecting differences in stimulation protocols and device design.

Table 1 and Figure 2 highlight the wide variability in tDCS device configurations, including differences in electrode montages, stimulation intensities, and target brain regions. Most studies utilized conventional 1 × 1 tDCS systems, particularly from Soterix Medical, with common electrode placements over the Dorsolateral Prefrontal Cortex (DLPFC). Current intensities ranged from 0.5 mA to 4 mA, indicating diverse therapeutic or experimental goals. Electrode sizes varied significantly, from small circular pads (3.14 cm^2^) to larger rectangular ones (5 × 7 cm^2^), influencing current density and focality. Multi-electrode and high-definition systems were used in some studies to achieve more targeted or distributed stimulation across cortical regions. Specially, Gurr et al.’s tDCS protocol applied Ruffini et al.’s Stimweaver algorithm to compute their optimized multichannel tDCS montage [16,27].

#### 3.1.2. Research Foci and tDCS Response Definitions

**Figure 3.**
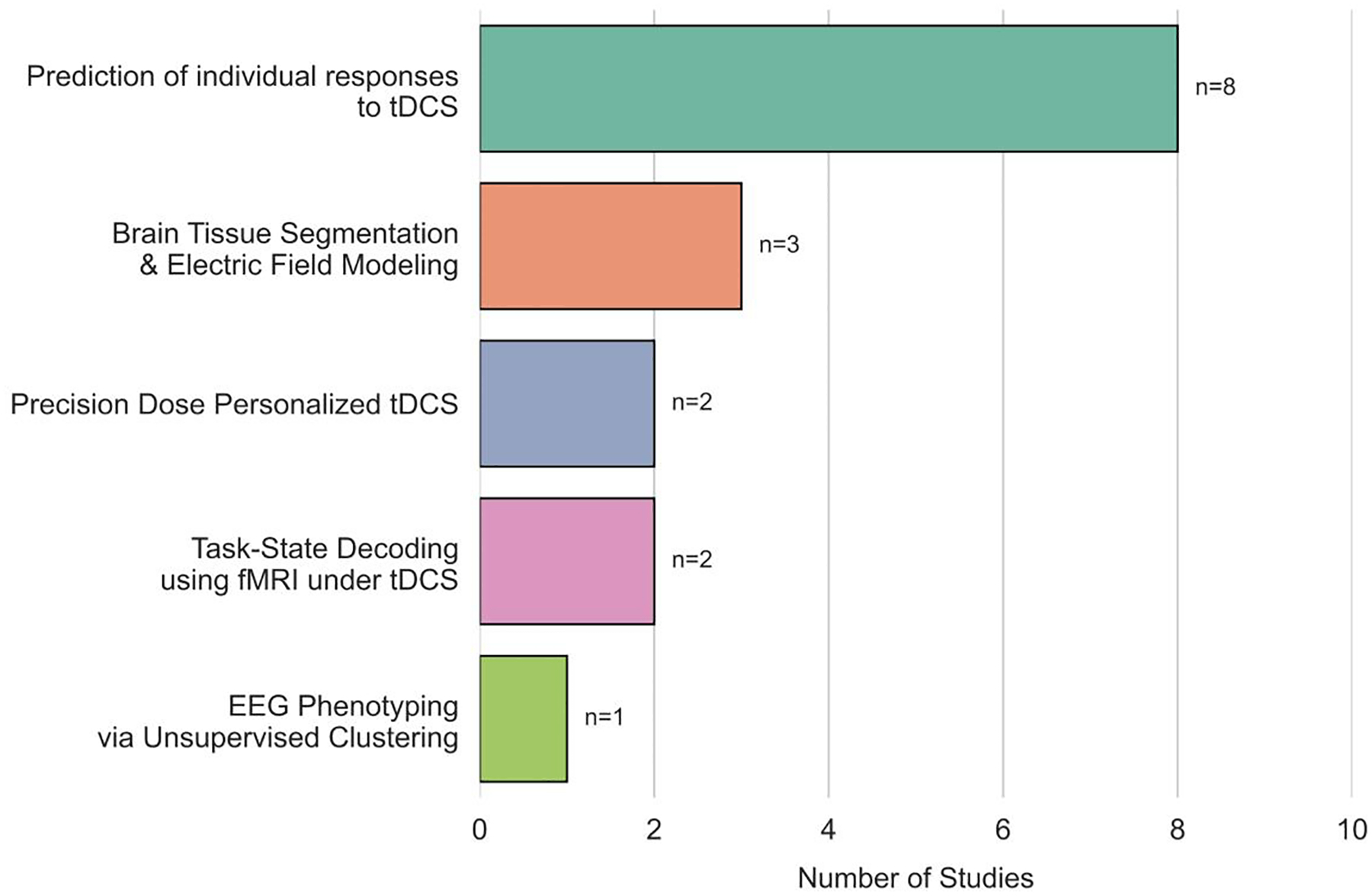
Distribution of studies by classification group. Each bar represents a task-based category in tDCS research; numbers (#) indicate the count of studies per group.

The ML application situation in reviewed tDCS studies is summarized in Table 2. Among the 16 reviewed tDCS studies, the primary research emphasis was on the prediction of individual responses to tDCS, with 8 studies (4 using EEG, 3 using neuroimaging, and 1 using demographics/clinical data) focused on forecasting tDCS related intervention response. Head tissue segmentation and electric field modeling (3 studies) and neuroimaging-driven tDCS personalization (2 studies) also emerged as key areas, targeting optimization of stimulation parameters through physiological and anatomical insights. Task-state decoding with fMRI was explored in 2 studies, highlighting the relevance of cognitive state classification during stimulation. Meanwhile, EEG-based phenotyping using unsupervised clustering appeared in 1 study, indicating a niche but growing interest in identifying data-driven EEG subtypes to stratify tDCS effects. These study categories are summarized in the bar chart shown in Figure 3. Taken together, this distribution illustrates an accelerating trend toward multimodal, personalization-oriented ML approaches that aim to translate tDCS from experimental paradigms toward clinically actionable, patient-specific interventions.

The definition of individual responses to tDCS in studies of prediction of individual responses varies across studies, depending on the specific clinical or physiological outcomes being targeted. This variability is reflected in the diverse criteria used to classify responders in ML applications. Some studies define responders based on clinical symptom improvement, such as remission from major depressive disorder (MDD) in Xiao et al.’s research [30], reduction in the Clinician-Administered Posttraumatic Stress Disorder Scale for DSM-5 (CAPS-5) in Kim et al.’s study [32], improved Coma Recovery Scale-Revised (CRS-R) scores in Zhang et al.’s study [33], or improved Auditory Hallucinations Subscale in Psychotic Symptom Rating Scales in Paul et al.’s study [38]. Cardon et al. defined responders to tDCS based on changes in the Tinnitus Functional Index (TFI) scores [39]. Gurr et al. and Albizu et al. measured changes in cognitive performance, such as improvements in neurocognitive task scores in participants after tDCS [16,37].

#### 3.1.3. Participant Populations and Demographics

**Figure 4.**
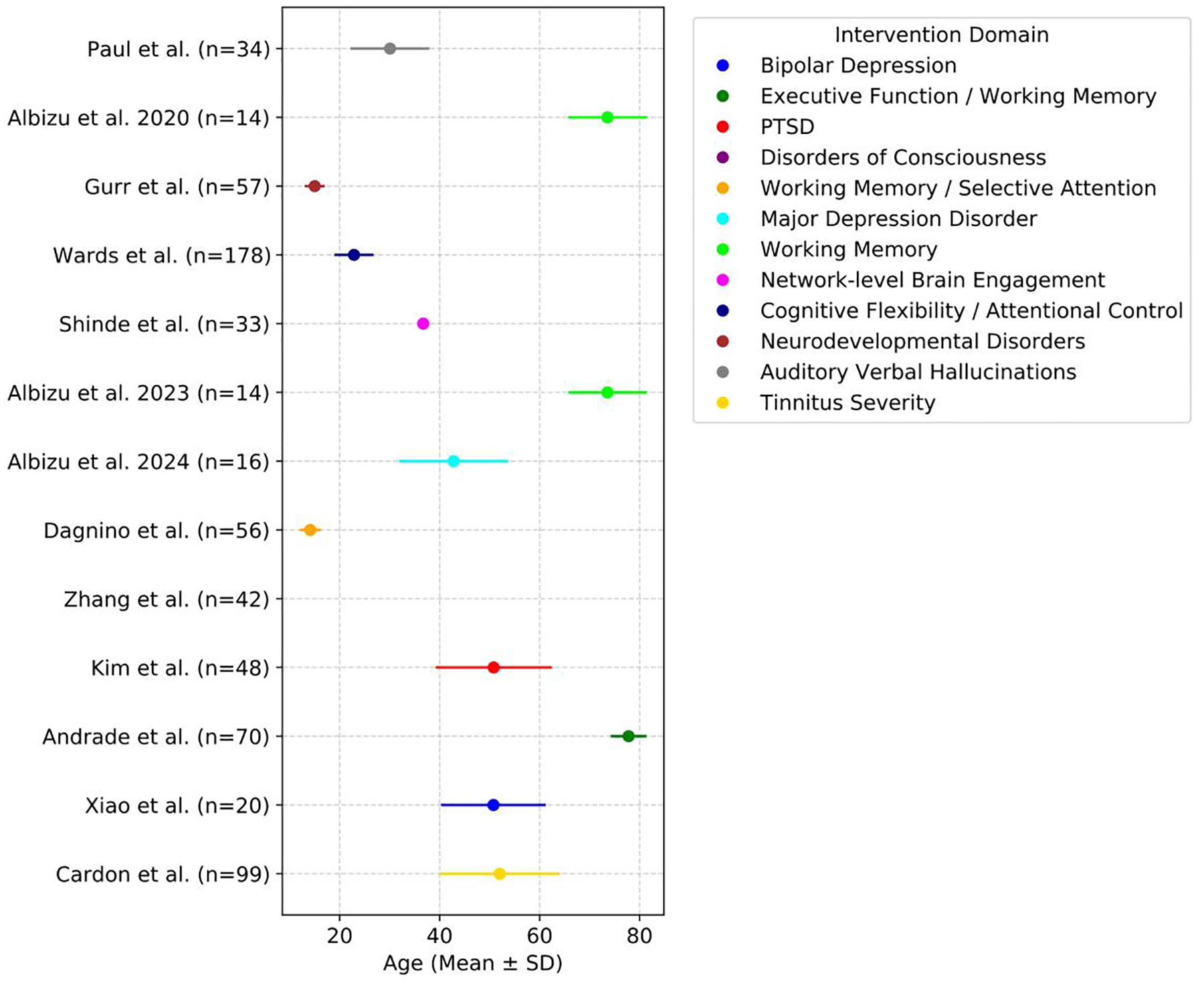
Participant age distribution across tDCS outcome prediction or EEG/fMRI analysis studies. This figure shows the mean age and standard deviation of participants across various studies (noting that Shinde et al. did not report age standard deviation, and the figure only shows its mean value). It highlights how different conditions are investigated across distinct age groups, ranging from children to older adults, with color coding indicating each study’s research focus. By visualizing the demographic context of the training data, the plot helps clarify the target domains and populations where the resulting ML models are likely to be applicable.

The reviewed studies show a diverse range of participant sources, highlighting the wide application of tDCS and ML across healthy individuals (7 papers) and various clinical populations (8 papers). Clinical populations include patients with AD, bipolar depression, MDD, Post-Traumatic Stress Disorder (PTSD), stroke, tinnitus, disorders of consciousness (DOC), and schizophrenia. The demographic profiles across the tDCS outcome-prediction and EEG/fMRI analysis studies (Table 3 and Figure 4) reveal substantial heterogeneity in therapeutic indication, age ranges, and sex distributions. This variability reflects the wide clinical and cognitive contexts in which tDCS is applied, but it also means that different age groups and populations effectively form distinct data domains, making it essential to evaluate how well predictive models generalize to out-of-domain samples. As ML methods continue to advance, effectively incorporating this demographic diversity will be essential for improving precision neuromodulation and tailoring tDCS to individual patients.

#### 3.1.4. Data Modalities

**Figure 5.**
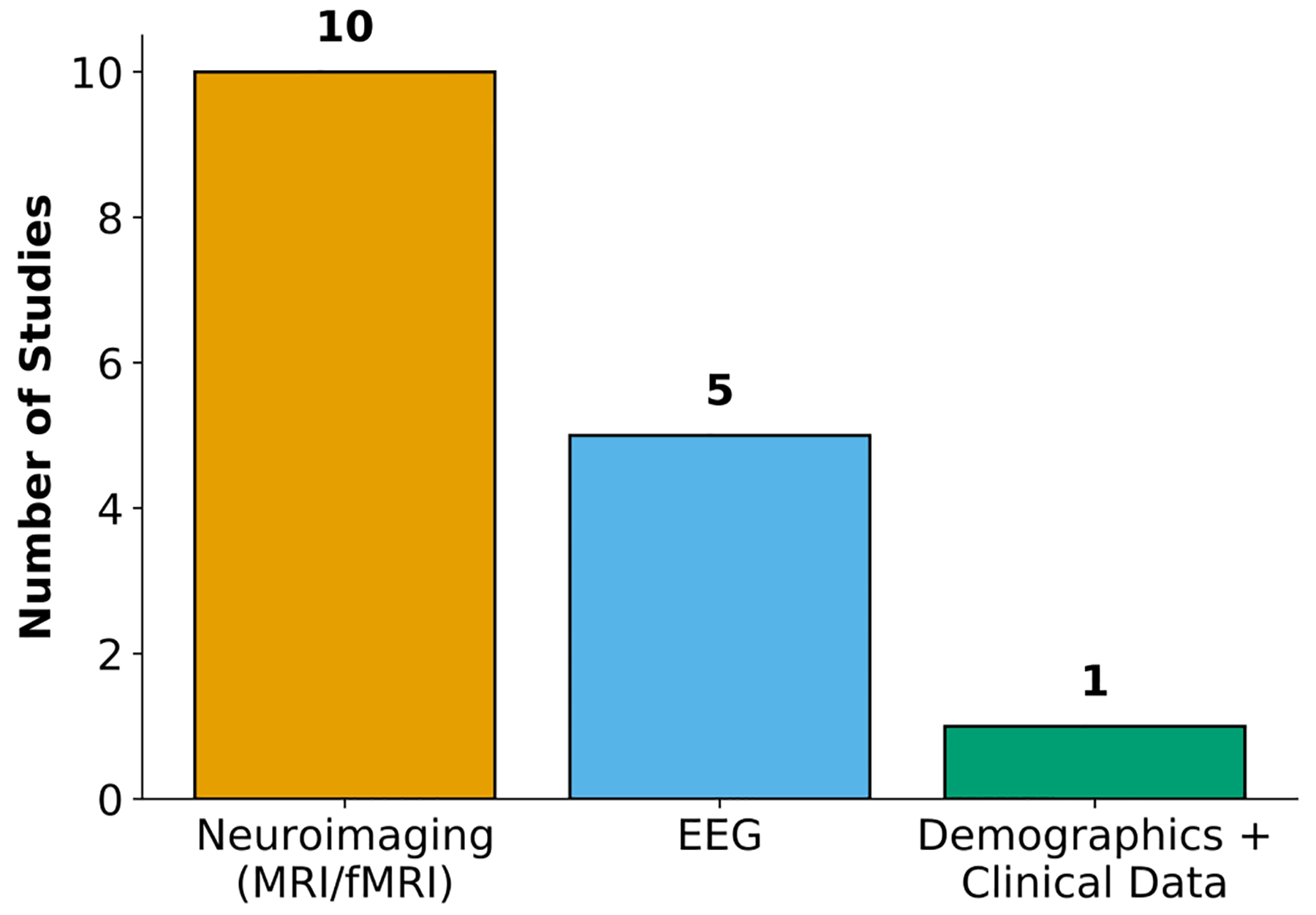
Distribution of Input Modality Usage Across Reviewed tDCS Studies, showing the proportion of studies using EEG-based inputs, MRI-based inputs, and demographics + clinical data.

The distribution of input modalities across the 16 reviewed tDCS studies highlights a predominant reliance on neuroimaging data. As shown in Figure 5, over half of the studies (10 out of 16) employed neuroimaging techniques such as structural magnetic resonance imaging (MRI) or fMRI, reflecting the field’s emphasis on structural and functional brain information to guide or interpret stimulation effects. EEG was the next most used modality, appearing in five studies, reflecting a strong interest in electrophysiological markers for response prediction and stratification. Additional EEG calculations included phase locking values, spectral power, normalized spatial complexity, and Welch power spectral density differences to capture brain activity patterns linked to model predictions. Only 1 study used demographics and clinical scores as predictors, suggesting that although these features are readily available, they are currently less commonly used for predicting tDCS outcomes. This distribution of input modalities underscores the growing importance of multimodal neuroimaging and electrophysiology in advancing precision neuromodulation.

### 3.2. Machine Learning Methods, Validation, and Software Tools

#### 3.2.1. ML Method Choices Across Studies

**Figure 6.**
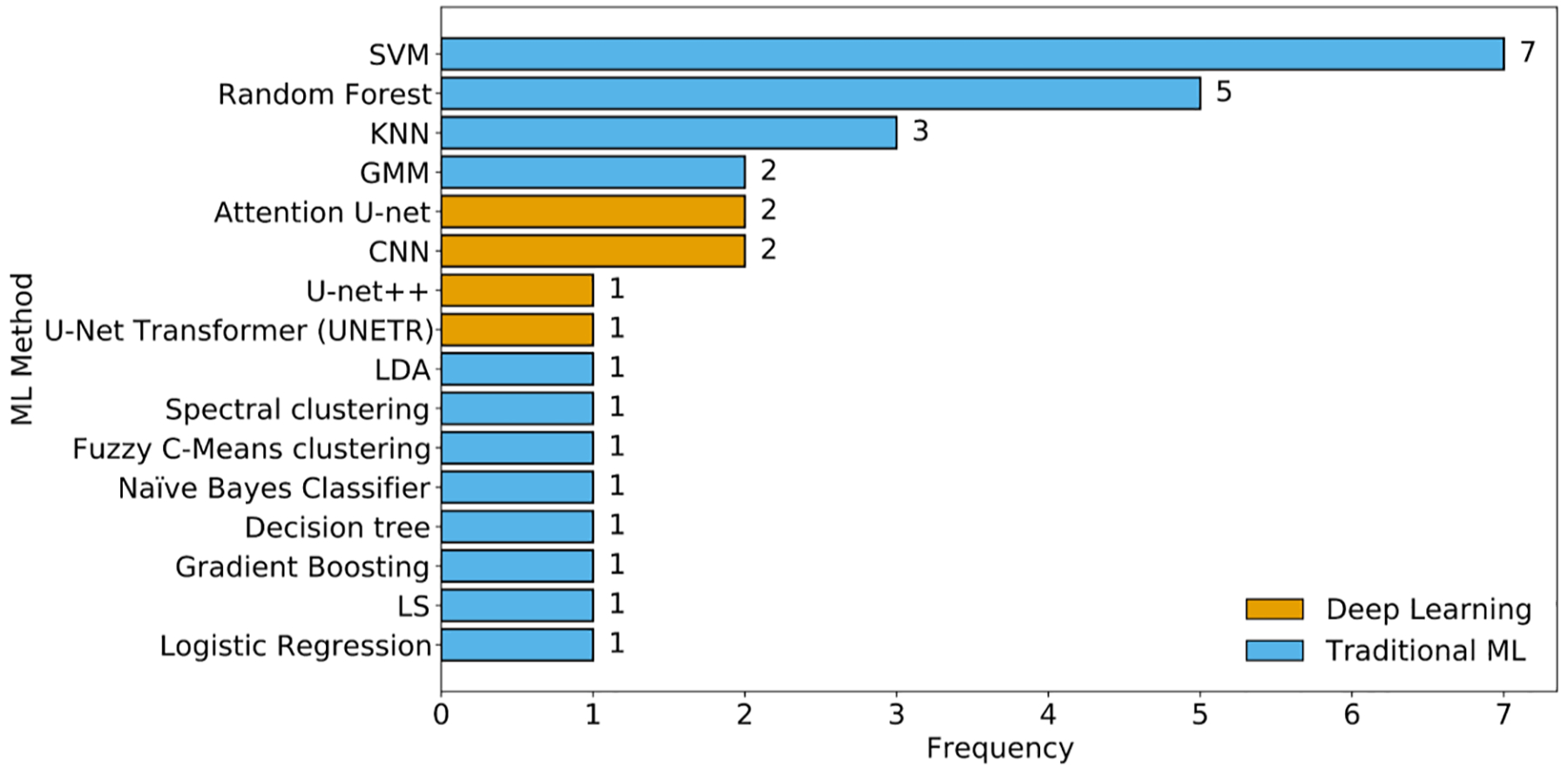
Frequency of ML methods employed across tDCS studies. Support vector machine (SVM) variants were the most frequently used, followed by Random Forest and K-Nearest Neighbors (KNN), reflecting a predominant reliance on traditional classifiers in tDCS research.

The bar chart in Figure 6 illustrates the frequency of ML methods used in tDCS-related studies. Support vector machine (SVM) emerges as the most commonly applied method, appearing in various forms (linear, and RBF kernel), highlighting its popularity in both classification and regression tasks. Random forest and K-nearest neighbors (KNN) also feature prominently, indicating their utility in handling diverse data types such as EEG, MRI, and clinical information. DL approaches like U-Net and its variants (including Attention U-Net and UNETR) are increasingly used for segmentation and current density estimation tasks, particularly in image-based studies. Clustering techniques such as spectral clustering and fuzzy C-means appear less frequently, primarily used for unsupervised stratification of EEG responses. This distribution highlights the widespread use of traditional ML classifiers in response prediction tasks, alongside the increasing interest in DL for imaging-related pattern recognition tasks.

#### 3.2.2. Data Splitting and Evaluation Metrics

Most studies used neuroimaging data and relied on cross-validation methods such as ten-fold and leave-one-out. EEG studies often applied subject-specific splits like leave-one-out cross-validation (LOOCV). Data splitting methods across reviewed studies are listed in Table 4. These studies evaluated model performance using metrics such as accuracy, sensitivity, specificity, and AUC to assess classification quality, along with the dice similarity coefficient (DICE) score, Hausdorff distance (HD), Average Symmetric Surface Distance (ASSD), precision, and recall for segmentation tasks. They also reported predicted and simulated response likelihoods to evaluate treatment and dose optimization, as well as variance explained in behavioral outcomes.

#### 3.2.3. Software Tools

A variety of specialized software tools listed in Table 5 are used across imaging and electrophysiological modalities to acquire, preprocess, and analyze data in tDCS research, ensuring accurate modeling of brain activity and stimulation effects.

### 3.3. Neuroimaging-Based Computational Modeling for tDCS

#### 3.3.1. Electrical Field and Current Density Distribution Modeling

Accurate estimation of electric field and current density distributions (J-maps) is foundational to understanding and optimizing tDCS effects, as these maps provide individualized insight into stimulation dose and neural engagement. The quasi-static form of Maxwell’s equations under the assumption of no time-varying fields and negligible displacement currents is a valid low-frequency approximation of tDCS [43]. Accurate electrical field distribution and J-map in a human head during tDCS intervention, based on precise tissue segmentation and the electrical and magnetic field theory, are important modalities in tDCS research. Software packages such as Simulation of Non-invasive Brain Stimulation (SimNIBS), Realistic vOlumetric-Approach-based Simulator for Transcranial electric stimulation (ROAST) generate individualized J-maps by integrating MRI-based tissue segmentation with electromagnetic field modeling, thereby enabling mechanistic interpretation of tDCS effects. Indahlastari et al. conducted the largest study to date using computational models (ROAST v2.7.1) to investigate tDCS field distribution in 587 healthy older adults [44]. Their findings show that brain atrophy, which increases with age, reduces the amount of tDCS current reaching the brain, highlighting the need to adjust stimulation parameters in aging populations.

The computational demands of computational modeling have catalyzed the adoption of DL methods, which offer substantial advantages in accelerating segmentation and current flow prediction while preserving anatomical fidelity. Additionally, DL methods can substantially accelerate the computational processes involved in producing individualized J-maps, thereby facilitating more efficient and scalable tDCS modeling for both research and clinical applications [40,42,45–47]. Jia et al. [40] introduced an Attention U-Net framework capable of real-time estimation of J-maps using MRI-derived volume conductor models (VCMs), marking a significant advance in the scalability of individualized tDCS modeling. The incorporation of attention mechanisms allowed the network to prioritize anatomically and functionally relevant structures, thereby improving spatial precision in current density predictions. Jia et al. used a loss function based solely on data, which may have multiple local minima, making optimization unreliable. Incorporating physics-informed regularization by combining the loss function could reshape the Jia et al.’s optimization landscape to better guide the model toward the global minimum [48], to enhance model training efficiency, leading to more generalized DL models for tDCS.

Together, these developments underscore the growing importance of combining data-driven modeling with biophysical knowledge to enable more reliable and scalable tDCS field prediction frameworks.

#### 3.3.2. Tissue Segmentation for Head Modeling

Recent developments in DL also advanced the accuracy and efficiency of neuroimaging tissue segmentation. Two important contributions in this area are the GRACE model by Stolte et al., designed for whole-head segmentation in older adults, and the fine-grained brain tissue segmentation framework by Lee et al., focused on stroke-induced abnormalities [41,42].

Stolte et al. proposed GRACE, a transformer-based UNETR model trained to segment 11 anatomically meaningful head tissues using T1-weighted MRI. The model was developed using 177 manually corrected scans and outperformed six existing tools and a 3D U-Net in terms of DICE score and HD. GRACE also segments a full head in under four seconds, making it suitable for fast, individualized head modeling workflows in aging populations.

In addition, Lee et al. developed a framework for brain tissue segmentation in stroke patients using advanced convolutional neural networks, including U-Net, U-Net++, HighResNet, and DeepLabv3. These models were enhanced with attention modules and novel convolution blocks, improving sensitivity to subtle or diffuse stroke lesions. The approach showed better performance than classical methods in delineating stroke-induced structural changes [41].

GRACE enables rapid, accurate whole-head modeling in older adults, crucial for finite element method-based current-flow calculation, while Lee et al.’s method improves lesion-specific segmentation in chronic stroke to enhance patient-specific modeling. In future work, several promising directions remain to be explored. Extending GRACE to include pathological brains, where lesions and atrophy may substantially alter current pathways, and expanding Lee et al.’s framework to incorporate extracranial tissues such as the skull and scalp would further enhance the fidelity of patient-specific models. Progress in these areas would help move the field toward more comprehensive, pathology-aware head models for clinically optimized tDCS planning.

### 3.4. Predictive Modeling Findings Across Data Modalities

#### 3.4.1. Overall Trends of Machine Learning Methods: From Hand-Crafted Features to Learned Representations

Recent advances in predictive modeling and EEG/fMRI analysis in tDCS research show a clear shift in how brain data are modeled. Earlier studies relied on interpretable, feature-driven ML models, while newer work is beginning to explore DL architectures that can model more complex brain dynamics.

Interpretability remains essential for clinical adoption because clinicians need transparent, biologically plausible explanations for model predictions. Traditional machine-learning models (e.g., Andrade; Kim; Wards; Shinde; Zhang; Gurr; Dagnino; Cardon) naturally provide this interpretability through feature weights, importance rankings, or anatomically grounded relevance maps [16,31–36,39]. In these approaches, researchers select predefined features such as EEG band power spectral density or functional connectivity so that model predictions can be directly linked to biologically meaningful mechanisms. In contrast, DL models do not rely on predefined features. Instead, they automatically learn hierarchical representations directly from the data, allowing them to capture complex spatial and temporal patterns. This ability improves modeling flexibility but reduces the transparency of how predictions are made.

Early DL efforts such as the work by Xiao et al. and Paul et al. [30,38] demonstrate the potential of pretrained backbones and transfer learning to improve tDCS response prediction. These approaches can extract spatial structure from MRI and temporal dynamics from EEG more effectively than hand-crafted features alone.

Despite these emerging strengths, a major methodological gap remains: there are no standardized, head-to-head comparisons of ML methods across the reviewed studies. Achieving balanced evaluations with consistent preprocessing and rigorous validation protocols is critical to reducing model-selection bias and enabling meaningful performance benchmarking.

#### 3.4.2. EEG-Based Predictors and Phenotypes

In EEG-based ML models, researchers commonly extract features like spectral power, phase locking values, and normalized spatial complexity to capture key aspects of brain activity. Xiao et al., Andrade et al., Kim et al., Zhang et al., and Dagnino et al. collectively demonstrate that frequency-specific EEG features, especially in the Delta, Theta, and Gamma bands, can effectively predict tDCS responsiveness across psychiatric conditions (e.g., bipolar depression, PTSD) and neurological disorders (e.g., AD, DOC) populations [30–34] as shown in Table 6.

For bipolar depression treated with home-based tDCS, Xiao et al.’s 1D Convolutional Neural Network (CNN) found that higher baseline phase locking values in theta, beta, and gamma bands predicted remission [30]. In AD, Andrade et al.’s random forest discovered that spectral power density in frontal and parietal-occipital regions (specifically electrodes FC1, F8, CP5, Oz, and F7 in EEG 10–10 system) predicted cognitive improvement defined by Alzheimer’s Disease Assessment Scale–Cognitive Subscale as an outcome of tDCS combined with cognitive intervention [31]. For DOC, Zhang et al.’s SVM with a linear kernel utilized normalized spatial complexity to show that alpha and gamma band organization predicted response defined by Coma Recovery Scale–Revised scores following posterior HD-tDCS [33].

In healthy pediatric populations, Dagnino et al. identified distinct “digital EEG phenotypes” in the alpha band from fuzzy-C clustering and spectral clustering on baseline EEG that predicted N-Back accuracy improvement after active stimulation [34]. Finally, for PTSD, Kim et al.’s SVM with RBF kernel demonstrated that spectral power densities, particularly in the delta band at midline and frontal sites (e.g., Cz, FC2 in EEG 10–10 system), effectively distinguished responders from non-responders defined by CAPS-5 scores to DLPFC-targeted tDCS [32].

#### 3.4.3. MRI-Based and Current Density-Based Predictors

**Figure 7.**
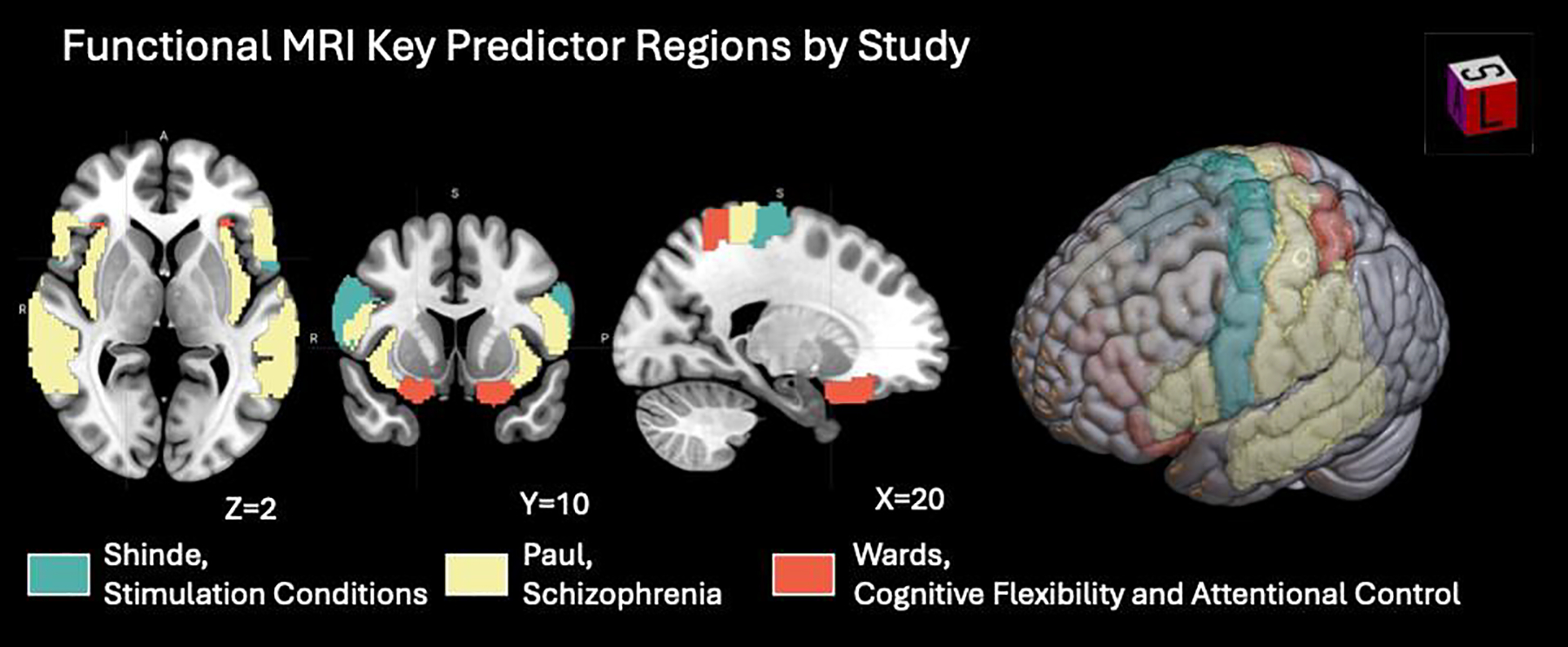
Functional MRI predictor regions across ML studies of tDCS and related therapeutic indications. Visualizations were generated using MRIcroGL v26.0.1 (https://www.nitrc.org/), with overlays mapped onto the MNI152 template supplied by the software.

**Figure 8.**
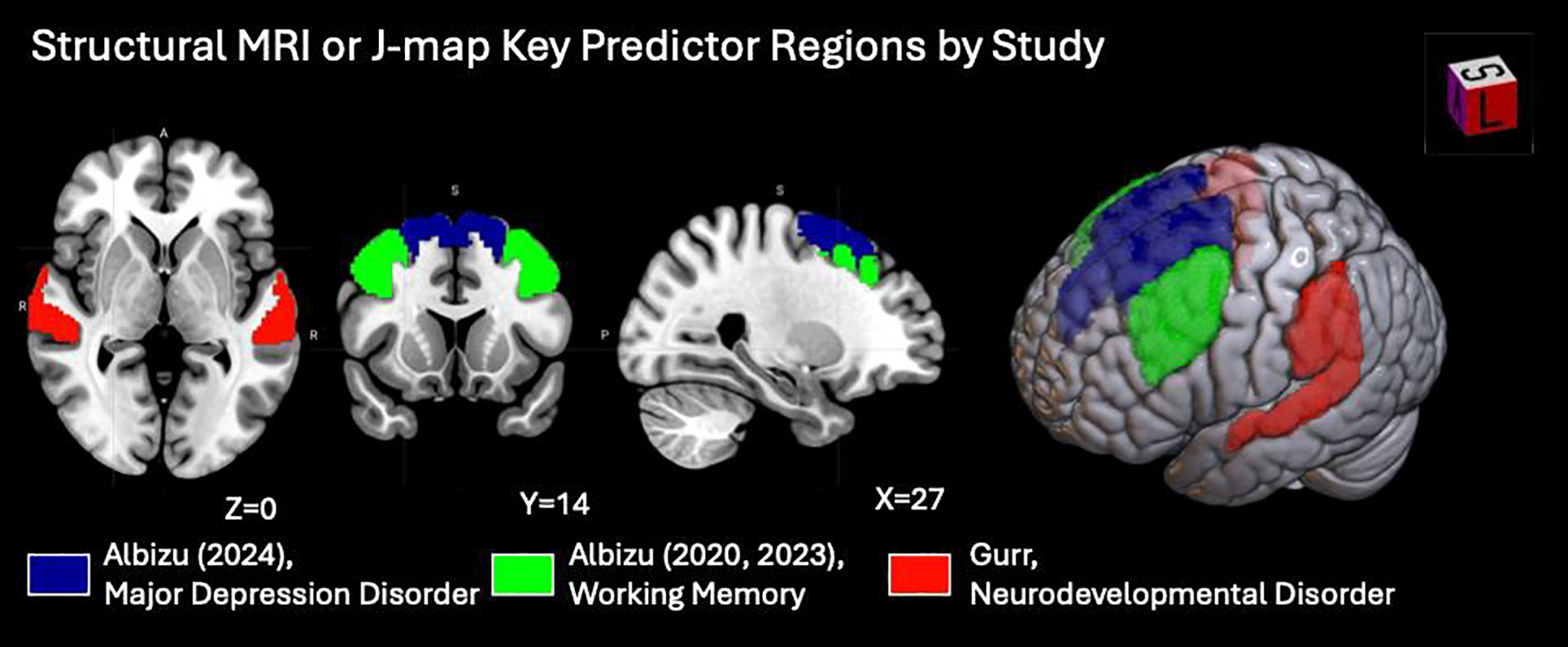
Structural MRI or J-map predictor regions across ML studies of tDCS and related therapeutic indications. Visualizations were generated using MRIcroGL v26.0.1 (https://www.nitrc.org/), with overlays mapped onto the MNI152 template supplied by the software. Albizu (2020, 2023) refers to two separate studies conducted by Albizu et al., published in 2020 and 2023, respectively.

In neuroimaging-based ML models, feature extraction typically involves deriving structural and functional metrics such as cortical thickness, white matter surface area, current density distribution, functional connectivity, and voxel-level activation. As shown in Table 7 and Figures 7 and 8, Shinde et al., Wards et al., and Paul et al. focused on fMRI features, dynamic connectivity, and task-related activation to capture brain network engagement relevant to tDCS outcomes. Gurr et al. extracted structural features to predict individual variability in treatment response. Across these studies, a common approach is the transformation of high-dimensional MRI data into interpretable, biologically meaningful features that align with cognitive or clinical effects of tDCS [16,29,37,38]. Uniquely among the reviewed studies, Albizu et al. leveraged T1-weighted MRI–derived J-maps to train ML models that predict tDCS responses in working memory and depression.

Research using fMRI highlighted the importance of network connectivity and neural representation overlap in predicting treatment effects. In schizophrenia, Paul et al.’s logistic regression model found that resting-state functional connectivity within the left superior temporal gyrus and its connections to the insula and motor cortices predicted improvement in auditory hallucinations after add-on tDCS [38]. Regarding cognitive multitasking in healthy adults, Wards et al. used SVM-based multivariate decoding to show that reduced decoding accuracy, indicating greater representational overlap in frontal and parietal regions, predicted visual search improvements following 1 mA left prefrontal tDCS [36]. Additionally, for targeted network engagement, Shinde et al. utilized random forest on dynamic functional connectivity during concurrent tDCS-fMRI to identify that multi-electrode stimulation yielded the clearest engagement of the Arcuate Fasciculus Network, with specific ROI pairs (e.g., Right Supplementary Motor Area-Right Inferior Precentral Gyrus) serving as strong predictors [35].

Gurr et al.’s study focusing on structural anatomy suggested that the physical dimensions of the cortex, distinct from cortical thickness, carry predictive weight for cognitive enhancement [16]. Gurr et al. utilized Partial Least Squares Regression (PLS) to demonstrate that individual differences in cortical surface area were the primary predictors of cognitive improvement in healthy adults. Specifically, using anodal tDCS over the left DLPFC, a larger surface area in the supramarginal gyrus and posterior superior temporal regions predicted better working memory improvement measured by N-back task accuracy difference. These findings indicate that the structural integrity of distributed networks, not just the tissue under the electrode, determines the efficacy of tDCS for cognitive tasks.

Albizu et al.’s pilot studies integrate J-map and ML models for tDCS response prediction. In the context of working memory, Albizu et al.’s SVM found that greater electric field intensity and directionality [37], particularly under and between the F3–F4 electrodes in EEG 10–20 system, predicted larger cognitive improvements. Expanding on this in the domain of depression, Albizu et al.’s SVM showed that current density magnitude across the brain, specifically in prefrontal and medial-temporal regions like the superior frontal gyri was the strongest separator of responders from non-responders defined by the 17-item Hamilton Depression Rating Scale [28]. Albizu et al.’s study further utilized these machine-learning weights to create a precision-dosing scheme that customized electrode placement to optimize current delivery for individual anatomies. Albizu et al.’s framework emphasized that the precise “dose” of electricity reaching specific brain tissues is a critical determinant of response.

#### 3.4.4. Clinical and Behavioral Predictors

Cardon et al.’s study demonstrated that advanced neuroimaging is not always necessary to predict outcomes, as standard clinical assessments can harbor significant predictive power. For patients with tinnitus who were treated with HD-tDCS, random forest in Cardon et al.’s study [39] found that baseline clinical metrics were the most effective predictors of response defined by TFI scores. Specifically, high scores on the baseline TFI and maximal perceived loudness were the key features distinguishing responders from non-responders. The model also identified depressive symptoms and handedness as influencing factors, suggesting that patients with higher initial distress and specific demographic profiles are more likely to respond favorably to neuromodulation targeting the right DLPFC.

### 3.5. Clinical Translation and Regulatory Maturity of ML-Enhanced tDCS

Based on Van Norman’s review [49], as a new medical device without a predicate, a tDCS system is initially Class III by default, but undergoes FDA review of the device, its intended use, and treatment protocol, and may pursue the De Novo pathway to obtain Class II classification by demonstrating reasonable assurance of safety and effectiveness under 21 CFR 860.7(d)–(e) [50].

As described in the medical device development framework presented by Kaplan et al. and Herrmann et al. [51,52], tDCS development must focus on the clinical-evaluation pathway. Early feasibility studies are required to (1) establish initial safety, usually in a small number of study subjects (15 or fewer based on [28], <100 based on [53]) for early clinical evaluation of devices to provide proof of principle and initial clinical safety data, (2) optimize stimulation parameters and study procedures, and (3) inform the design of later-stage trials. Once feasibility is demonstrated, and if pivotal trials are required, we must design pivotal trials to (4) demonstrate safety and effectiveness in the intended population using FDA-aligned endpoints and Good Clinical Practice (GCP) standards, typically through a large, multicenter study involving hundreds to more than 1000 participants across 20–50 sites, depending on the risk profile and indication. All 13 tDCS studies out of the 16 reviewed tDCS studies that used ML about tDCS intervention outcome prediction or EEG/fMRI analysis were based on data from early-feasibility/pilot clinical trial or preclinical/exploratory human research, as shown in Table 8.

### 3.6. Methodological Limitations and Opportunities for Deep Learning Advancement in tDCS Research

Among the 16 included studies, only five have explored DL approaches, especially those using pretrained models or transfer learning. These methods could be particularly valuable for tDCS research, as they help overcome small-sample limitations and capture richer physiological patterns. With the emergence of pretrained models and transfer learning frameworks, it is now possible to leverage knowledge learned from extensive neuroimaging and physiological datasets to better recognize complex patterns in new tDCS clinical trial data and to enhance model performance even with limited samples.

Due to the large volume of available literature and resource constraints, we employed a pragmatic Google Scholar–based search strategy, as used in Peralta et al.’s survey work [54]. Reliance on Google Scholar’s relevance-based ranking may introduce selection bias by preferentially surfacing highly cited or positive studies. Combined with the restriction to English-language, peer-reviewed publications, this approach may overlook relevant but less visible or unpublished studies with null or negative results. Consequently, the final pool of included papers may overrepresent successful or high-impact findings, introducing a potential publication bias in the review.

## 4. Future Directions

Before outlining these future directions, we first examine the potential sources of bias and limitations inherent in our literature search and study selection process, as well as the methodological constraints observed across the 16 reviewed studies. Based on these limitations, this section identifies several promising avenues for future research, including the incorporation of richer physiological data modalities, the use of physical and biophysical modeling, multimodal data integration, and adaptive learning frameworks. In particular, future work should emphasize precision tDCS interventions, with more accurate control of stimulation parameters such as current intensity, timing, and waveform, as well as optimized and individualized electrode placement and montage design, to better target specific neural circuits. These advances are expected to improve both the predictive accuracy of outcome models and the clinical relevance and reproducibility of tDCS-based interventions.

### 4.1. Precision and Multimodal tDCS Clinical Trial Design

Across multiple therapeutic indications, tDCS, both as a standalone intervention and in combination with other interventions (such as cognitive training or behavioral therapy), remains a potential alternative or complement to pharmacological solutions. For example, reports from the National Academies of Sciences, Engineering, and Medicine emphasize that for Alzheimer’s disease and Alzheimer’s disease–related dementias (AD/ADRD), non-pharmacological interventions continue to be promising and clinically relevant, particularly given limited disease-modifying drug efficacy [53]. Within this framework, tDCS is recognized as a scalable, low-risk neuromodulation approach that can be integrated into multimodal treatment strategies targeting cognitive, behavioral, and functional outcomes.

Given the expanding application of tDCS across multiple therapeutic indications, future clinical trials must move beyond one-size-fits-all designs and explicitly address heterogeneity in clinical targets, brain states, and treatment responses. Future tDCS trials should therefore be designed to (1) accommodate therapeutic and response heterogeneity by supporting modality-specific outcome and responder definitions across cognitive, affective, and behavioral domains and by enabling stratification based on baseline brain and physiological state rather than excluding mixed or nonuniform response profiles; (2) improve inclusion, diversity, and representativeness by reducing participation burden through scalable and remote-capable protocols and ensuring generalizability across diagnoses, symptom severity, demographic characteristics, and comorbidity profiles; and (3) rigorously evaluate multimodal tDCS interventions using adaptive or factorial designs that test stimulation in combination with cognitive training, behavioral therapy, or pharmacological treatments across distinct therapeutic indications. In parallel, strengthening infrastructure for longitudinal and real-world tDCS evaluation through continuous physiological data collection, harmonized data pipelines, and trial designs capable of capturing long-term and population-level variability is essential to fully characterize inter-individual differences and sustained treatment effects that are not observable in short-duration, single-endpoint studies.

Achieving these goals requires the integration of objective biomarkers and adaptive optimization frameworks that reflect the dynamic nature of neuromodulation. Specifically, (1) integrating validated physiological and neurobiological biomarkers into tDCS trials can improve trial efficiency and interpretability by characterizing baseline brain state, confirming target engagement, refining responder classification, and detecting treatment effects earlier and more objectively than symptom-based measures alone. In parallel, (2) adopting adaptive, control-theoretic, and biophysically informed trial frameworks for tDCS could enable dynamic optimization of stimulation parameters including current dose, electrode montage, stimulation timing, and session scheduling under uncertainty while respecting safety and physiological constraints, where ML–based models have potential to support personalized policy adaptation, state estimation, and decision-making within these adaptive trial designs.

### 4.2. Physiological Data Modalities

Recent studies demonstrate the feasibility of using physiological time-series data to model psychiatric states and treatment response using ML. Such signals may be particularly valuable for predicting individual variability in tDCS outcomes, as they capture moment-to-moment physiological changes before, during, and after stimulation. However, physiological recordings collected during tDCS can be contaminated by stimulation-related artifacts, requiring careful preprocessing and validation to ensure reliable interpretation.

Physiological signals (e.g., electrocardiogram, EEG, electrodermal activity, photoplethysmography) serve as primary sensor outputs, which are then transformed into clinically interpretable physiological metrics such as heart rate variability, respiration rate, sleep patterns, and stress markers. These metrics offer a structured bridge between raw sensor data and psychiatric criteria, enabling ML models to learn temporal patterns associated with symptom severity and treatment response.

Finally, the choice of ML approach should align with data modality and scale. Traditional ML methods remain effective for structured physiological features derived from time-series data, particularly when interpretability and limited sample sizes are critical. DL approaches are better suited for raw, high-dimensional, and multimodal time-series data (e.g., EEG, audio, video), where modeling temporal dependencies and cross-modal interactions is essential. Focusing on continuous physiological data collection and time-series ML modeling is therefore a key step toward more objective, individualized, and predictive tDCS outcome assessment.

### 4.3. Toward a Control Framework for tDCS Optimization

Building on the expanded role of psychiatric and physiological data modalities discussed above, an important next step is to formalize tDCS optimization within a stochastic control framework, where psychiatric measurements serve as observable (albeit noisy) indicators of latent brain states. Such a formulation enables precision tDCS interventions by treating stimulation parameters, including current dose, electrode placement, stimulation timing, and session scheduling, as controllable inputs that can be adaptively adjusted to optimize clinical and cognitive outcomes over time.

Albizu et al. conducted two studies using the same SVM + Gaussian Mixture Model (GMM)-based dose optimization method, differing only in cognitive function and MDD [28,29]. While this demonstrates methodological consistency, they treated dose-response as static and their control objectives relied on the working memory and depression measurement at post-intervention, so that the duration of treatment effect using their optimization method is still unknown, because they did not continuously collect longitudinal data. As their dataset lacked time-series recordings and their studies were based on secondary analysis, they did not model how brain states evolve over time steps; consequently, the sequential decision-making and long-term planning are absent. In contrast, Gebodh et al.’s study presents an advancement with a dynamic, closed-loop neuromodulation framework [55]. Their use of ML to predict responsiveness and guide stimulation decisions marks a shift toward adaptivity. Although none of them focused on long-term reward optimization or planning, their system avoids trial-and-error learning, unlike reinforcement learning methods such as TD-learning, Q-learning, or policy gradient methods, due to safety concerns. While emphasizing flexibility and real-time responsiveness, this limitation hinders refinement of stimulation policies based on longitudinal responses to tDCS interventions. Thus, their framework advances practical implementation of current control of tDCS but stops short of leveraging full control-theoretic and reinforcement learning potential.

Overall, existing studies demonstrate the feasibility of using psychiatric measurements to inform tDCS parameter selection, but they fall short of exploiting the full potential of control theory and stochastic optimization. A principled stochastic control formulation integrating psychiatric assessments with physiological signals could enable precision tDCS by jointly optimizing current dose, electrode montage, stimulation timing, and session scheduling under uncertainty. Such an approach would support long-term planning, safety-aware policy learning, and personalized intervention strategies, ultimately improving the robustness, efficacy, and clinical relevance of tDCS treatments.

### 4.4. Biophysically Constrained Models for Adaptive tDCS

A promising direction in tDCS research is the integration of biophysically informed models that incorporate physical laws, such as Maxwell’s equations, used to compute electric fields into model training. This approach enhances current flow estimation and supports personalization by addressing key challenges like limited generalizability, static dose-response assumptions, and inter-individual anatomical variability.

For example, Mellot et al. introduce a physics-informed, unsupervised, and source-free domain adaptation framework for harmonizing EEG data across heterogeneous datasets with varying electrode configurations [56]. Their key contribution is a field interpolation (FI) method that maps EEG signals to a fixed sensor layout using a forward model grounded in quasi-static approximation of Maxwell equations, current dipole model, and minimum norm estimation (MNE) in the EEG inverse problem, summarized by Baillet et al. [57]. Evaluated on six motor imagery brain-computer interface (BCI) datasets with Riemannian geometry-based classification, FI outperforms alternative imputation and interpolation methods, especially when there are few shared channels across datasets. FI’s basis in biophysical modeling, use of canonical head models, ability to reconstruct signals at arbitrary locations, and goal of harmonizing across electrode layouts suggest it could be adapted to account for variability in tDCS montages and individual head anatomies. Building on this trend of integrating physical models into neural data analysis, Morik et al. [58] introduced 3D-PIUNet, a hybrid EEG source localization framework that refines a physics-informed pseudo-inverse solution (eLORETA) using a 3D convolutional U-Net. By combining the strengths of classical models and data-driven learning, it achieved superior spatial accuracy and robustness across diverse synthetic and real-world EEG settings. Morik et al.’s approach could similarly enhance tDCS research by improving the precision of brain activity mapping and enabling better evaluation of stimulation effects.

### 4.5. Transfer and Contrastive Learning and Multimodality Integration

Future research on tDCS treatment outcome prediction could benefit from leveraging pretrained DL models. DL–based tDCS outcome prediction may leverage diverse neurophysiological and neuroimaging inputs such as structural MRI for anatomical features, fMRI for dynamic brain activity, and EEG for electrophysiological signals. Transfer and contrastive learning help integrate these complementary modalities.

In the structure MRI domain, recent advances in transfer learning have opened new opportunities for improving the efficiency of structure MRI data in neuroscience and neuroimaging research. As demonstrated by KrishnaPriya et al., transfer learning with pre-trained CNNs such as VGG-19 can achieve high accuracy in brain structure MRI classification even with limited data [59]. Their work highlights the potential of reusing learned representations from large-scale neuroimaging datasets to enhance performance in smaller, domain-specific tasks.

In the fMRI domain, unlike data in the fields of computer vision (with ImageNet) or NLP (with large text corpora), fMRI datasets are typically expensive to acquire and highly variable across scanners, protocols, and preprocessing pipelines, which makes it especially difficult to build robust, large-scale pretrained models. This scarcity and heterogeneity severely limit opportunities for transfer learning, which is crucial for overcoming small sample size constraints in downstream tasks. Recent advances such as NeuroSTORM by Wang et al. (pre-trained on over 50,000 subjects and 28.65M fMRI frames), BrainLM by Ortega Caro et al. (trained on 6700 h of fMRI recordings), and BrainGFM by Wei et al. (pre-trained across 27 datasets, 25,000 subjects, and 400,000 graph samples) demonstrate the potential of foundation models to enable effective transfer learning by providing general-purpose, transferable fMRI representations [60–62].

Similarly, in the EEG domain, challenges such as high dimensionality and variability across recording devices and protocols hinder model generalizability and limit transfer learning opportunities. Junhong Lai’s recent survey of EEG foundation models highlights emerging resources such as EEGPT, Neuro-GPT, and CBraMod, which aim to build scalable, generalizable EEG representations that can support transfer learning across tasks and datasets [63].

Additionally, advances in multimodal models such as CLIP from Radford et al.’s work [64], which aligns heterogeneous data types like images and text in a shared embedding space, offer a promising foundation for integrating structure to support more precise, personalized treatment strategies.

## 5. Conclusions

This review underscores the potential of ML to address the fundamental limitation of tDCS research: large inter-individual variability under uniform stimulation protocols. Emerging ML-based approaches shift tDCS from region-centric and polarity-based assumptions toward individualized, network-level modeling that links anatomy, brain state, and delivered dose to outcomes. However, most existing studies remain exploratory, relying on small sample sizes, limited external validation, and underutilization of advanced ML or DL frameworks. Future research should prioritize prospective multimodal trials, biophysically constrained modeling of electric fields, and adaptive, control-oriented frameworks that allow stimulation parameters to be optimized over time. Leveraging transfer learning and foundation models will also be critical for improving generalizability across populations and devices. Together, these directions define a pathway toward precision neuromodulation, where tDCS interventions are personalized, mechanism-aware, and clinically scalable.

## Figures and Tables

**Table 1.** Summary of tDCS Device Usage Across 16 Studies.

Study	Device	Cathode/Anode Location (Montage)	Current through Electrodes	Electrode Specification
**Albizu et al. (2024) [** 28 **]**	Conventional 1 × 1 tDCS (Soterix Medical)	Anode: Left DLPFC (F5), Cathode: Right DLPFC (F6), per 10–20 EEG system	2.0 m A	5 × 5 cm^2^
**Albizu et al. (2023) [** 29 **]**	Conventional 1 × 1 tDCS (Soterix Medical)	F3 (cathode) and F4 (anode), per 10–20 EEG system	2.0 mA	5 × 7 cm^2^
**Xiao et al. [** 30 **]**	Flow Neuroscience tDCS device	F3 (anode) and F4 (cathode), per 10–20 EEG system	2.0 mA	Electrode Area 23 cm^2^
**Andrade et al. [** 31 **]**	TCT-Research neurostimulator	Six cortical areas affected by AD, F3, F5, CP5, P3, per 10–20 EEG system	2.0 mA	4 × 4 cm^2^
**Kim et al. [** 32 **]**	Not Specified	F3 (anode) and F4 (cathode), per 10–20 EEG system	2.0 mA	Not Specified
**Zhang et al. [** 33 **]**	Soterix Medical Inc.	Cathodes: CPz, POz, P3, P4; Anode at Pz per 10–20 EEG system	2.0 mA	Not Specified
**Dagnino et al. [** 34 **]**	Soterix Medical Inc.	Left DLPFC and right IFG	2.0 mA	Electrode Area 3.14 cm^2^ (circular)
**Shinde et al. [** 35 **]**	Neuroconn DCMC stimulator	Posterior STG/MTG, SMG, posterior IFG (SE & ME montages)	4 mA (SE); 2.0 mA, 1.0 mA, 1.0 mA (ME)	Not Specified
**Wards et al. [** 36 **]**	Neuroconn DCMC stimulator	Anode 1 cm posterior to F3 or F4; cathode over supraorbitofrontal cortex (mirrored setup)	1.0 mA (LH, RH); 2.0 mA (LH)	5 × 5 cm^2^
**Gurr et al. [** 16 **]**	The Starstim 32 stimulator	AF3, AF7, F3, Fp2, T7; IFGright: F8, FC6, P7, T8, C6, FPz; TPJbilateral: CP5, CP6, C5, P1, PO7, C4, T8, P6	897 μA, 284 μA, 819 μA, −1000 μA, −1000 μA etc.	Electrode Area 3.14 cm^2^ (circular)
**Albizu et al. (2020) [** 37 **]**	Conventional 1 × 1 tDCS (Soterix Medical)	F3 (cathode) and F4 (anode), per 10–20 EEG system	2.0 mA	5 × 7 cm^2^
**Paul et al. [** 38 **]**	Soterix Medical MxN-HD system	CP5 (−2 mA), FC3, FT7, PO7, P1 (+0.5 mA each).	−2.0 mA (center), +0.5 mA each (returns)	4 × 1 multichannel stim. adaptor, Outer radius: 12 mm, Inner radius: 6 mm (Annular)
**Cardon et al. [** 39 **]**	1 × 1 HD-tDCS (Soterix Medical)	Central anode at F4; cathodes at AF4, FC4, F6, F2 (10–20 EEG system)	2.0 mA	4 × 1 multichannel stim. adaptor, Outer radius: 12 mm, Inner radius: 6 mm (Annular)

**Table 2.** Summary of 16 Studies on Machine Learning Applications in tDCS Research.

Study	Therapeutic Indication	Dataset	ML Method	Clinical Target	ML Task	Key ML Result
**Albizu et al. (2024) [** 28 **]**	MDD	16 MDD patients’ T1 MRIs (current density distribution)	SVM + GMM	Predict responders defined by HDRS-17 depression scores, and Optimize tDCS dosing	Classify responders, personalize dose	Post-optimization: 100% reclassified as responders
**Albizu et al. (2023) [** 29 **]**	Working memory (cognitive function)	14 healthy older adults’ T1 MRIs (current density distribution)	SVM + GMM	Optimize tDCS dosing	Classify responders, personalize dose	Post-optimization: 100% reclassified as responders
**Jia et al. [** 40 **]**	Not applicable	85 subjects structural MRIs (SimNIBS output as ground truth), Latin hypercube sampling applied, 85,000 samples generated.	Attention U-Net	Estimate tDCS-induced current density	Predict 3D current density map	Achieved near real-time estimation
**Lee et al. [** 41 **]**	Chronic stroke	580 subjects in the chronic stage of stroke, 580 T1 MRIs	Attention U-Net, U-Net++	Stroke brain segmentation	Segment tissues, lesions	DICE: 0.55, HD 12.94, ASSD 3.83
**Stolte et al. [** 42 **]**	Not applicable	177 healthy older adults’ T1 MRIs	U-Net transformer (UNETR)	Whole-head tissue segmentation	Segment tissues	DICE: 0.89, HD: 2.87
**Xiao et al. [** 30 **]**	Bipolar depression	20 bipolar depression patients, EEG (Phase Locking Value)	1D CNN	Predict clinical remission	Classify responders	Accuracy 69%, sensitivity 76%
**Andrade et al. [** 31 **]**	Executive function, Working memory (cognitive function)	70 AD patients, EEG (Spectral power)	Random forest	Predict response to tDCS defined by ADAS-Cog	Classify responders	Top EEG features: FC1, F8, CP5; accuracy: 72%
**Kim et al. [** 32 **]**	PTSD	48 PTSD patients, EEG (Power Spectral Density)	SVM (RBF)	Predict response to tDCS defined by CAPS-5 scores	Classify responders	Best AUC 0.93 (SVM, delta band Power Spectral Density)
**Zhang et al. [** 33 **]**	DOC	42 DOC patients, EEG (normalized spatial complexity of multi-channel EEG data)	SVM (linear), LDA, random forest, KNN	Predict response to HD-tDCS defined by CRS-R scores	Classify responders	Best accuracy 93%, AUC 0.9 (SVM)
**Dagnino et al. [** 34 **]**	Working memory, Selective Attention, etc. (cognitive function)	A total of 206 samples were collected from 56 children, each of whom underwent 4 EEG sessions following data cleaning, EEG (relative band power)	Spectral clustering, Fuzzy C-Means clustering	Stratify EEG activity response to tDCS (N-back, Flanker task, and Continuous Performance Task)	Cluster EEG phenotypes, the clusters derived from FCM were used to correlate EEG-based cluster memberships with behavioral performance differences (active vs. sham tDCS) in cognitive tasks.	Four distinct clusters of participants were identified (Silhouette = 0.26, Davies Buoldin = 1.2, Calinski Harabasz = 92, when the number of clusters is four, in Spectral clustering)
**Shinde et al. [** 35 **]**	Network-level brain engagement in right arcuate fasciculus network	33 participants, rs-fMRIs (dynamic functional connectivity matrices)	KNN, random forest, naïve Bayes classifier, decision tree, gradient boosting	Explore tDCS montage effects	Classify ME/SE/NS conditions	Best accuracy 92% (KNN)
**Wards et al. [** 36 **]**	Cognitive flexibility, attentional control (cognitive function)	178 adults, fMRIs (task-related activation of each voxel based on general linear model)	Conduct the subsequent multivariate pattern analysis (MVPA) using linear SVM	Identify whether a brain activity pattern corresponds to an auditory or visual task	Predict the task condition (auditory vs. visual single-task)	Decoding accuracy drop in specific ROIs predicted faster visual search after left PFC tDCS
**Gurr et al. [** 16 **]**	Neurodevelopmental disorders (cognitive function)	57 children, structural MRIs (aggregated Cortex thickness, surface area of white matter of region of interest)	LS, SVM, random forest, KNN, PCR, PLS, and ICR	Predict tDCS-induced neurocognitive task score changes	Regressor predict individual response in terms of changes in neurocognitive task performance	R^2^ = 0.652, RMSE = 8.34 (DLPFC left target region, difference in accuracy in N-back task, PLS)
**Albizu et al. (2020) [** 37 **]**	Working memory (cognitive function)	14 healthy older adults, structural MRIs (current density distribution)	SVM	Predict response to tDCS defined by N-back task score	Classify responders	Averaged overall accuracy of 86.43%
**Paul et al. [** 38 **]**	Persistent auditory verbal hallucinations in Schizophrenia	34 Schizophrenia patients with auditory verbal hallucinations, rs-fMRIs (correlation values to selected region of interest)	Logistic regression, CNN	Predict response to tDCS defined by PSYRATS-AH score	Classify responders	Best accuracy: 72.5% (Logistic regression over the LSTG connectivity features with voxels in brain regions implicated in AVH pathophysiology)
**Cardon et al. [** 39 **]**	Tinnitus severity	99 tinnitus patients, demographics + clinical data	Random forest	Predict response to tDCS defined by TFI score	Classify responders	Accuracy 85.7%, sensitivity 100%

**Table 3.** Participants’ demographics across tDCS outcome prediction or EEG/fMRI analysis studies.

Study	Therapeutic Indication	Age (Mean ± Standard Deviation)	Sex
**Xiao et al. [** 30 **]**	Bipolar depression	50.75 ± 10.46	Not specified
**Andrade et al. [** 31 **]**	Executive function, working memory (cognitive function)	77.8 ± 3.6	32 females, 38 males
**Kim et al. [** 32 **]**	PTSD	50.81 ± 11.60	25 females, 23 males
**Zhang et al. [** 33 **]**	DOC	Not specified	Not specified
**Dagnino et al. [** 34 **]**	Working memory, selective attention, etc. (cognitive function)	14.09 ± 2.1	32 females, 24 males
**Albizu et al. (2020 and 2023) [**29,37**]**	Working memory (cognitive function)	42.8 ± 10.9	7 females, 7 males
**Albizu et al. (2024) [** 28 **]**	MDD	73.57 ± 7.84	9 females, 7 males
**Shinde et al. [** 35 **]**	Network-level brain engagement in right arcuate fasciculus network	36.7 (Standard deviation is not revealed)	14 females, 19 males
**Wards et al. [** 36 **]**	Cognitive flexibility, attentional control (cognitive function)	22.86 ± 3.93	119 females, 59 males
**Gurr et al. [** 16 **]**	Neurodevelopmental disorders (cognitive function)	15 ± 2	34 females, 23 males
**Paul et al. [** 38 **]**	Persistent auditory verbal hallucinations in schizophrenia	30.06 ± 7.89	15 females, 19 males

**Table 4.** Summary of Dataset Characteristics, Data Modalities, and Model Validation Strategies Across Reviewed tDCS-Related ML Studies.

Study	Samples in Dataset	Data Modality	Data Splitting
**Albizu et al. (2024) [** 28 **]**	16 MDD patients	T1 MRI (Current density maps derived from T1 MRIs)	Eight-fold cross-validation
**Albizu et al. (2020 and 2023) [**29,37**]**	14 healthy older adults	T1 MRI (Current density maps derived from T1 MRIs)	Seven-fold cross-validation
**Jia et al. [** 40 **]**	85 subjects structural MRIs (Latin hypercube sampling applied, 85,000 samples generated.)	T1 MRI	Hold-out
**Lee et al. [** 41 **]**	580 subjects in the chronic stage of stroke	T1 MRIs	Hold-out
**Stolte et al. [** 42 **]**	177 healthy older adults	T1 MRIs	Hold-out
**Xiao et al. [** 30 **]**	20 bipolar depression patients	EEG (phase locking values)	LOOCV
**Andrade et al. [** 31 **]**	70 AD patients	EEG (spectral power)	Three-fold cross-validation
**Kim et al. [** 32 **]**	48 PTSD patients	EEG (power spectral density)	Five-fold cross-validation
**Zhang et al. [** 33 **]**	42 DOC patients	EEG (normalized spatial complexity)	LOOCV
**Dagnino et al. [** 34 **]**	56 children (each of whom underwent 4 EEG sessions, a total of 206 samples were collected)	EEG (Relative band power)	M-hold-out using 80% for parameter search
**Shinde et al. [** 35 **]**	33 health adults	rs-fMRIs (dynamic functional connectivity matrices)	Ten-fold cross-validation
**Wards et al. [** 36 **]**	178 health adults	Task-based fMRIs (task-related activation of each voxel based on general linear model)	LOOCV
**Gurr et al. [** 16 **]**	57 neurotypical children	T1 MRIs (aggregated Cortex thickness, surface area of white matter of region of interest)	LOOCV
**Paul et al. [** 38 **]**	34 schizophrenia patients with auditory verbal hallucinations	rs-fMRIs (correlation values to selected region of interest)	Five shuffled iterations of 10-fold balanced cross-validation
**Cardon et al. [** 39 **]**	99 tinnitus patients	Demographic + clinical data	Five-fold cross-validation

**Table 5.** Summary of software used in reviewed tDCS research.

Analysis Category	Software	Primary Function	Primary Programming Language(s)
Structural MRI & Electric Field Modeling	dcm2niix	Converts MRI data from DICOM to NIfTI format	C/C++
	FreeSurfer	Head tissue segmentation and cortical surface reconstruction	C/C++, Tool Command Language (TCL), Python
	SimNIBS	Simulates electric fields in the brain using the finite element method	Python, C++, MATLAB (interfaces)
	ROAST	Realistic modeling of transcranial electric stimulation	MATLAB
	iso2mesh	Generates 3D finite element meshes from segmented volumes	MATLAB/Octave
	getDP	Solves finite element problems (e.g., electric field propagation)	C/C++
fMRI Analysis	CONN Toolbox (v18b)	Preprocessing and connectivity analysis using aCompCor	MATLAB
	fMRIPrep (v20.2.3)	Standardized preprocessing for anatomical and functional MRI	Python
	SPM12	Voxel-wise univariate analysis using GLM	MATLAB
	MarsBaR	ROI-based analysis integrated with SPM	MATLAB
	PRoNTo (v3)	Multivariate pattern analysis (MVPA) for classification tasks	MATLAB
	GRETNA	Graph-theoretical analysis of brain networks from functional connectivity data	MATLAB
EEG Processing	BrainVision Analyzer	Proprietary software for EEG acquisition and initial inspection	C++
	EEGLAB	EEG preprocessing and analysis toolbox in MATLAB	MATLAB
	MNE-Python	EEG/MEG processing in Python	Python

**Table 6.** Overview of EEG feature Bands, ML Models, and Performance Metrics in tDCS outcome prediction and EEG analysis studies.

Study	Therapeutic Indication	Best Band Combination for ML Models	ML Algorithm with Best Performance	Best Performance
**Xiao et al. [** 29 **]**	Bipolar depression	Theta/Beta/Gamma	1D CNN	Accuracy 69%
**Andrade et al. [** 30 **]**	Executive function, working memory (cognitive function)	Frontal Alpha/Theta	Random Forest	Accuracy 72%
**Kim et al. [** 31 **]**	PTSD	Delta	SVM	Accuracy 82%
**Zhang et al. [** 33 **]**	DOC	Gamma	SVM	Accuracy 93%
**Dagnino et al. [** 34 **]**	Working memory, selective attention (cognitive function)	Alpha	Spectral & FCM Clustering	Silhouette = 0.26, Davies Buoldin = 1.2, Calinski Harabasz = 92, when the number of clusters is four.

**Table 7.** Neuroimage-derived brain region and network predictors supporting ML Models of tDCS Response.

Study	Therapeutic Indication	MRI Modality	Brain Atlas Used	Key Brain Regions and Network Features Used as Predictors	ML Method with Best Performance	Best Performance
**Albizu et al. (2024) [** 28 **]**	MDD	T1 weighted MRI derived J-map	Harvard-Oxford	Left Superior Frontal Gyrus, Right Superior Frontal Gyrus, Right Supplementary Motor Area, etc.	SVM (linear)	Accuracy 91.25%
**Albizu et al. (2020 and 2023) [**29,37**]**	Working memory (cognitive function)	T1 weighted MRI derived J-map	Harvard-Oxford	Right Superior Frontal Gyrus, Left Superior Frontal Gyrus, Right Middle Frontal Gyrus, etc.	SVM (linear)	Accuracy 86.43%
**Shinde et al. [** 35 **]**	Network-level brain engagement in right arcuate fasciculus network	rs-fMRI	Harvard-Oxford	Arcuate Fasciculus Network nodes (Right Supplementary Motor Area-Right Inferior Precentral Gyrus)	KNN	Accuracy 92%
**Wards et al. [** 36 **]**	Cognitive flexibility, attentional control (cognitive function)	Task-based and resting-state fMRI and T1 weighted MRI	Not applicable	Orbitofrontal Cortex, Superior Parietal Lobe, Inferior Parietal Lobe, Cerebellar Vermis VI	SVM	Not revealed
**Gurr et al. [** 16 **]**	Neurodevelopmental disorders (cognitive function)	T1 + T2 weighted MRI	Desikan-Killiany	Left Supramarginal Gyrus, Left Superior Temporal Sulcus, etc.	PLS	RMSE: 8.34 R^2^: 0.652 MAE: 7.08
**Paul et al. [** 38 **]**	Persistent auditory verbal hallucinations in schizophrenia	rs-fMRI	Harvard-Oxford	Left and Right Superior Temporal Gyrus, Left Insular Cortex, Postcentral Gyrus, Precentral Gyrus, Right Inferior Frontal Gyrus, Left Middle Temporal Gyrus, Left Supramarginal Gyrus	Logistic regression	Accuracy 72.5%

**Table 8.** Clinical trial profiles and predictive findings in reviewed tDCS Studies.

Study	Therapeutic Indication	Type of Clinical Trial	Sample Size	Open-Label	Multi-Arm	Findings
**Albizu et al. (2024) [** 28 **]**	MDD	Early-feasibility/pilot clinical study	16	No	No	Personalized electric-field modeling showed that higher prefrontal and medial-temporal current density predicted which patients would respond to tDCS for depression.
**Albizu et al. (2023) [** 29 **]**	Working memory (cognitive function)	Early-feasibility/pilot clinical study	14	No	No	Greater modeled electric-field intensity and directionality beneath and between F3–F4 predicted larger working-memory improvements following tDCS.
**Xiao et al. [** 30 **]**	Bipolar depression	Early-feasibility/pilot clinical study	20	Yes	No	Higher baseline theta, beta, and gamma phase-locking values predicted clinical remission following home-based tDCS in bipolar depression.
**Andrade et al. [** 31 **]**	Executive function, Working memory (cognitive function)	Early-feasibility/pilot clinical study	70	No	Yes	Baseline spectral power density in frontal and parietal-occipital EEG regions predicted cognitive gains after combined tDCS and cognitive training in AD.
**Kim et al. [** 32 **]**	PTSD	Preclinical/exploratory human research	48	Yes	No	Spectral power patterns especially delta-band activity at midline and frontal sites reliably distinguished responders from non-responders to DLPFC-targeted tDCS.
**Zhang et al. [** 33 **]**	DOC	Preclinical/exploratory human research	42	Yes	No	Alpha and gamma band normalized spatial complexity features predicted recovery capacity following posterior HD-tDCS stimulation.
**Dagnino et al. [** 34 **]**	Working memory, Selective Attention, etc. (cognitive function)	Preclinical/exploratory human research	56	No	Yes	Distinct baseline EEG “digital phenotypes” predicted behavioral accuracy and reaction-time changes in children receiving active tDCS.
**Shinde et al. [** 35 **]**	Network-level brain engagement in right arcuate fasciculus network	Early-feasibility/pilot clinical study	33	Yes	No	Dynamic functional-connectivity features during stimulation especially between Supplementary Motor Area and inferior precentral regions were the strongest predictors of targeted network engagement.
**Wards et al. [** 36 **]**	Cognitive flexibility, attentional control (cognitive function)	Preclinical/exploratory human research	178	No	Yes	Greater representational overlap in frontal and parietal regions predicted improved visual-search performance after left prefrontal tDCS.
**Gurr et al. [** 16 **]**	Neurodevelopmental disorders (cognitive function)	Preclinical/exploratory human research	57	No	Yes	Individual differences in cortical surface area, rather than thickness, predicted the magnitude of behavioral improvement across working-memory and perceptual-decision tDCS tasks.
**Albizu et al. (2020) [** 37 **]**	Working memory (cognitive function)	Early-feasibility/pilot clinical study	14	No	No	Higher electric-field intensity under F3–F4 predicted which individuals would show the stronger working-memory improvements from anodal tDCS.
**Paul et al. [** 38 **]**	Persistent auditory verbal hallucinations in Schizophrenia	Preclinical/exploratory human research	34	Yes	No	Resting-state connectivity in the left superior temporal gyrus and its linked networks predicted reductions in auditory hallucinations after tDCS.
**Cardon et al. [** 39 **]**	Tinnitus severity	Early-feasibility/pilot clinical study	131	Yes	No	Baseline clinical measures, particularly TFI severity and perceived loudness, were the strongest predictors of symptom improvement following HD-tDCS.

## Abbreviations

AD: Alzheimer’s Disease
ADAS-Cog: Alzheimer’s Disease Assessment Scale–Cognitive Subscale
ASSD: Average Symmetric Surface Distance
AUC: Area Under the Curve (receiver operating characteristic analysis)
AVH: Auditory Verbal Hallucination
CAPS-5: Clinician-Administered PTSD Scale for DSM-5
CNN: Convolutional Neural Network
CRS-R: Coma Recovery Scale–Revised
DCMC: Direct Current Multi-Channel
DICE: Dice Similarity Coefficient
DL: Deep Learning
DLPFC: Dorsolateral Prefrontal Cortex
DOC: Disorders of Consciousness
DSM-5: Diagnostic and Statistical Manual of Mental Disorders, Fifth Edition
EEG: Electroencephalography
fMRI: Functional Magnetic Resonance Imaging
GMM: Gaussian Mixture Model
HD: Hausdorff Distance
HDRS-17: 17-item Hamilton Depression Rating Scale
HD-tDCS: High-Definition Transcranial Direct Current Stimulation
ICR: Independent Component Regression
IFG: Inferior Frontal Gyrus
J-map: Current Density Distributions
KNN: K-Nearest Neighbors
LDA: Linear Discriminant Analysis
LH: Left Hemisphere
LS: Least Squares Method
LSTG: Left Superior Temporal Gyrus
ME: Multi-Electrode
MDD: Major Depressive Disorder
ML: Machine Learning
MRI: Magnetic Resonance Imaging
MTG: Middle Temporal Gyrus
MVPA: Multivariate Pattern Analysis
NS: No Stimulation
PCR: Principal Component Regression
PFC: Prefrontal Cortex
PLS: Partial Least Squares Regression
PSYRATS-AH: Psychotic Symptom Rating Scales-Auditory Hallucinations Subscale
PTSD: Post-Traumatic Stress Disorder
R2: Coefficient of Determination
RBF: Radial Basis Function
REM: Rapid Eye Movement
RH: Right Hemisphere
RMSE: Root Mean Square Error
ROI: Region of Interest
rs-fMRI: Resting-State Functional Magnetic Resonance Imaging
SE: Single Electrode
SMG: Supramarginal Gyrus
SNR: Signal-to-Noise Ratio
STG: Superior Temporal Gyrus
SVM: Support Vector Machine
T1 MRI: T1-Weighted Magnetic Resonance Imaging
fMRI: Functional Magnetic Resonance Imaging
tDCS: Transcranial Direct Current Stimulation
TFI: Tinnitus Functional Index
TPJbilateral: Bilateral Temporoparietal Junction
U-Net/U-Net++: Deep Learning Architectures for Image Segmentation
UNETR: U-Net Transformer
